# Removal of growth hormone receptor (GHR) in muscle of male mice replicates some of the health benefits seen in global GHR−/− mice

**DOI:** 10.18632/aging.100766

**Published:** 2015-06-29

**Authors:** Edward O. List, Darlene E. Berryman, Yuji Ikeno, Gene B. Hubbard, Kevin Funk, Ross Comisford, Jonathan A. Young, Michael B. Stout, Tamar Tchkonia, Michal M. Masternak, Andrzej Bartke, James L. Kirkland, Richard A. Miller, John J. Kopchick

**Affiliations:** 1 Edison Biotechnology Institute, Ohio University, Athens, OH 45701, USA; 2 Department of Specialty Medicine, Heritage College of Osteopathic Medicine, Ohio University, Athens, OH 45701, USA; 3 School of Applied Health Sciences and Wellness, Ohio University, Athens, OH 45701, USA; 4 Department of Biomedical Sciences, Heritage College of Osteopathic Medicine, Ohio University, Athens, OH 45701, USA; 5 The Barshop Institute for Longevity and Aging Studies, San Antonio, Department of Pathology, The University of Texas Health Science Center at San Antonio, Research Service, Audie L. Murphy VA Hospital (STVHCS), San Antonio, TX 78229, USA; 6 Robert and Arlene Kogod Center on Aging, Mayo Clinic, Rochester, MN 55905, USA; 7 Department of Internal Medicine, Geriatrics Research, Southern Illinois University School of Medicine, Springfield, IL 62702, USA; 8 College of Medicine, Burnett School of Biomedical Sciences, University of Central Florida, Orlando, FL, 32827, USA; 9 Department of Head and Neck Surgery, The Greater Poland Cancer Centre, Poznan, 61-866, Poland; 10 Department of Pathology and Geriatrics Center, University of Michigan, Ann Arbor, MI 48109, USA

**Keywords:** growth hormone receptor (GHR), muscle-specific knockout, lifespan, aging, frailty, pathology, body composition

## Abstract

Global disruption of the GH receptor in mice (GHR−/−) produces a large and reproducible extension in lifespan. Since lack of GH action in muscle resulting in improved glucose homeostasis is potentially a mechanism by which GHR−/− mice are long-lived, and since no information on muscle-specific GHR disruption in females is available, we generated and characterized a line of muscle-specific GHR disrupted (MuGHRKO) mice. As expected, male MuGHRKO mice had improved fasting blood glucose, insulin, c-peptide, and glucose tolerance. In contrast, female MuGHRKO mice exhibited normal glucose, insulin, and glucose tolerance. Body weight was mildly but significantly altered in opposite directions in males (decreased) and females (increased) compared to controls. Grip strength and treadmill endurance were unchanged with advanced age in both sexes, suggesting that the direct action of GH on muscle has minimal effect on age-related musculoskeletal frailty. Longevity was unchanged in both sexes at Ohio University and significantly increased for males at University of Michigan. These data suggest that removal of GHR in muscle of male MuGHRKO mice replicates some of the health benefits seen in global GHR−/− mice including improvements to glucose homeostasis and smaller body weight in males, which may explain the trends observed in lifespan.

## INTRODUCTION

Mice with deficits in GH action have proven useful for investigating the effects of GH on aging. These mice are long-lived and show delays in many age-dependent changes, suggesting that they may be aging at a slower rate than controls [1-4]. In 1997, our laboratory developed GH receptor gene disrupted mice (GHR−/−) [5] in order to generate a mouse line specifically targeting GH action (as opposed to Ames and Snell dwarf mice with multiple pituitary deficits). Because GHR is absent in all tissues, GH action is eliminated with the other pituitary hormonal systems intact, making this mouse line a valuable tool for understanding the various actions of GH. Like Snell and Ames dwarf mouse lines, global GHR−/− mice are long-lived, with increased longevity being observed at several institutions, in both sexes, and in multiple background strains [6]. Studies by our laboratory and others have found that global GHR−/− mice are resistant to the following: cognitive decline [7, 8], musculoskeletal frailty [9], age-related accumulation of senescent cells [10], high fat diet induced hyperglycemia and hyperinsulinemia [11], diabetic nephropathy [12], and cancer [13-15]. Moreover, enhanced insulin sensitivity appears to be a prominent phenotype of these mice, which may partially explain their increased longevity [16-20].

To delineate the effects of GH on specific tissues, our laboratory and others have produced tissue-specific GHR gene disrupted (GHRKO) mice. Liver-specific deletion of GHR results in severely decreased circulating IGF-1 (~90%) with elevated GH (~300%), insulin resistance, hepatic steatosis [21, 22], alterations to most IGFBP's, increased adiponectin, and decreased body size (weight and length), despite increased local IGF-1 mRNA in extrahepatic tissues (such as skeletal muscle and adipose tissue) [22]. Tissue-specific disruption of GHR in pancreatic beta cells impairs insulin secretion, and this effect is exacerbated by a high fat diet [23]. Macrophage-specific deletion of GHR produces mice that are similar to controls with no changes to glucose or insulin tolerance with standard chow [24]. However, when placed on a high fat diet, mice lacking GHR in macrophages show impaired glucose and insulin tolerance as well as increased macrophage abundance, expression of pro-inflammatory cytokines, and crown like structures, which are indicative of dead or dying adipocytes and remodeling of adipose tissue [24]. Furthermore, conditioned media from macrophages cultured from these mice have increased inhibitory effect on adipogenesis [25]. Adipose tissue-specific disruption of GHR results in obesity with uniform increases in all adipose depots and normal glucose homeostasis [26]. These mice also have decreased levels of adipsin but normal levels of resistin and adiponectin (unlike the increased levels found in global GHR−/− mice), suggesting that GH does not regulate these adipokines directly in adipose tissue [26]. Two separate laboratories have published data on muscle-specific GHR gene disrupted mice and have shown significant effects on glucose homeostasis although the two groups reported opposite results [27-29]. Mavalli et al. [27] report increased adiposity with insulin resistance and glucose intolerance in muscle-specific GHR disrupted mice. In contrast, Vijayakumar et al. report reduced adiposity and overall improvement in glucose homeostasis [28, 29]. These disparities clearly justify further clarification of the role of muscle-specific GHRKO on glucose homeostasis. In addition, the previously published studies of muscle-specific GHR gene disrupted mice are limited to male mice and provide no insight into effects of muscle-specific GH disruption in females. Indeed, of the thirteen published reports utilizing tissue-specific GHR gene disrupted mice, only four compare males to females. Of the four studies that compared males to females, three found significant phenotypic changes that are sex-specific. For example, when GHR is deleted in liver, hepatic steatosis is limited to males [22], xenobiotic metabolizing enzyme mRNAs are oppositely regulated in males compared to females [30], and apoptosis-related mRNA's are decreased in female brain and kidneys but not in males [31]. Thus, these studies highlight the importance of utilizing both sexes when evaluating the effect of tissue-specific deletion of GHR.

No studies to date have assessed longevity or cause of death in muscle-specific GHR gene disrupted mice. Since glucose homeostasis has a strong influence on longevity, and since improved insulin sensitivity is thought to be, in part, responsible for the extended longevity in global GHR−/− mice, it was of interest to evaluate glucose homeostasis and lifespan in these mice. We hypothesize that loss of direct GH action on muscle contributes to the enhanced longevity in global GHR−/− mice. To test this hypothesis, we generated a line of muscle-specific GHR gene disrupted (MuGHRKO) mice to examine longevity and cause of death. We also determined the effects of muscle-specific GHR gene disruption on the GH/IGF-1 axis, glucose homeostasis, body composition, energy expenditure, and musculoskeletal frailty in both sexes.

## RESULTS

### Creation of MuGHRKO mice

**Figure 1:**
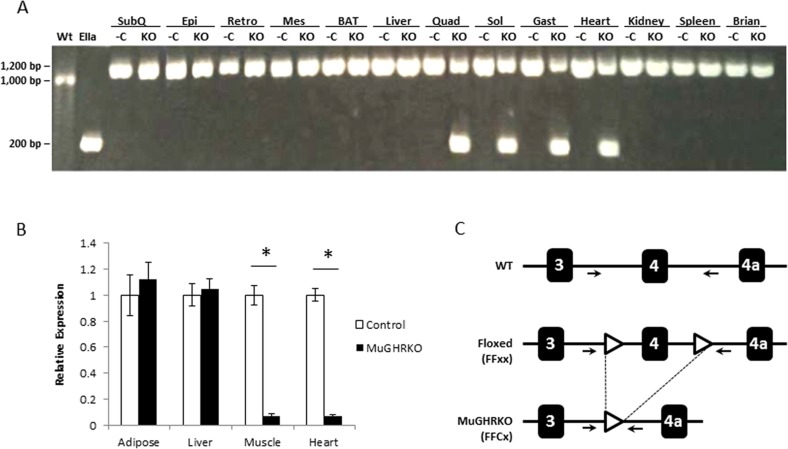
Disruption of GHR gene in muscle (**A**) DNA isolated from WAT (subcutaneous or SubQ, epididymal or Epi, retroperitoneal or Retro, and mesenteric or Mes depots), BAT, liver, skeletal muscle (quadriceps or Quad, gastrocnemius or Gast, and soleus or Sol), heart, kidney, spleen, and brain tissues was analyzed by PCR. The negative control (first lane on the left) is DNA isolated from liver of floxed control mice (WT), while the positive control (second lane on the left) is DNA isolated from the liver of global EIIaGHRKO mice. PCR primer locations are shown in part C of this figure. For each tissue, floxed controls (-C) are on the left while MuGHRKO (KO) are on the right. (**B**) Quantification of GHR mRNA levels in WAT, liver, skeletal muscle (quadriceps), and heart in floxed controls (n=6; white bars) and MuGHRKO mice (n=6; black bars) was performed by real time RT-PCR. (**C**) Schematic representation of Cre-Lox mediated disruption of the GHR gene. Primer locations (used for PCR in part A of this figure) are indicated with arrows. * indicates significant difference at p<0.05.

MuGHRKO mice and floxed controls were generated by breeding conditional GHR^flox/flox^ mice to muscle-specific Cre-recombinase transgenic mice, B6.FVB(129S4)-Tg(Ckmm-cre)5Khn/J. For MuGHRKO mice, tissue specificity for disruption of the GHR gene was detected in skeletal muscle (quadriceps, soleus, and gastrocnemius muscles) and heart as determined by PCR (Fig 1A). No GHR gene disruption was detected in other tissues, including white adipose tissue WAT (subcutaneous, epididymal, retroperitoneal, mesenteric depots), brown adipose tissue (BAT), liver, heart, kidney, spleen, and brain. Quantification by real time RT-PCR showed >95% reduction in GHR mRNA in skeletal muscle (quadriceps) and heart tissues (Fig 1B). No change in GHR gene expression was observed in WAT or liver.

### Measures of GH, IGF-1 and IGF binding proteins in MuGHRKO mice

**Figure 2:**
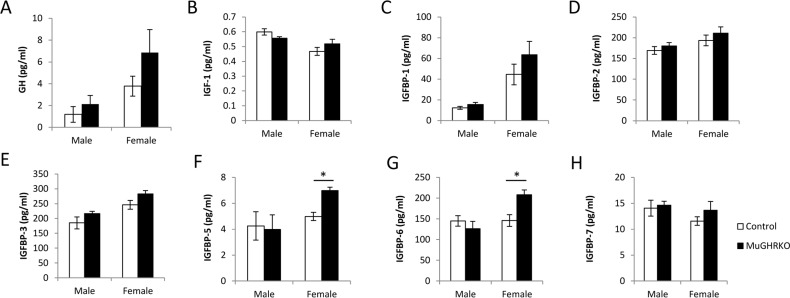
Effects of muscle-specific disruption of GHR on the GH/IGF-1 axis (**A**) GH, (**B**) IGF-1, and (**C**-**H**) IGFBP levels are shown for male and female MuGHRKO (black bars) and controls (white bars). * indicates significant difference at p<0.05.

To test the effects of muscle-specific disruption of the GHR on the GH/IGF-1 axis, serum levels of GH, IGF-1 and IGPBP-1, -2, -3, -5, -6 and -7 were measured at 6 months of age (n=9-10, per group, per sex). Serum GH and IGF-1 levels were unchanged in MuGHRKO compared to controls for both sexes (Fig 2A & B). No significant changes were observed for IGFBP-1, -2, -3, or -7 (Fig 2C-E, H) in either sex. Serum levels of IGFBP-5 (Fig 2F) and IGFBP-6 (Fig 2G) were significantly increased in female MuGHRKO mice but not in males. No significant changes were seen in IGF-1 mRNA levels in MuGHRKO mice compared to controls in liver, white adipose tissue, brown adipose tissue, heart, or skeletal muscle (data not shown). Thus, removal of direct GH action on muscle has no measurable effect on IGF-1 with regards to circulating IGF-1 or IGF-1 mRNA in various tissues, including muscle and heart; however, IGFBP-5 and -6 are significantly increased in females.

### Measures of glucose homeostasis and adiponectin in MuGHRKO mice

**Figure 3:**
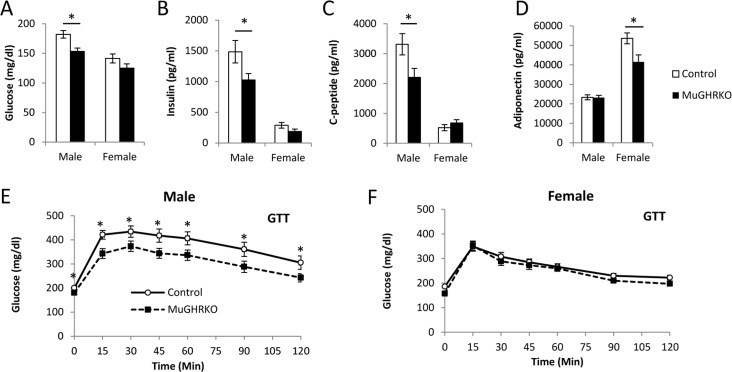
Measures of glucose homeostasis in male and female MuGHRKO mice (**A**) Fasting blood glucose, (**B**) insulin, (**C**) c-peptide, and (**D**) adiponectin levels are shown for male and female MuGHRKO (black bars) and controls (white bars). Glucose tolerance tests (**E**-**F**) are shown for male and female MuGHRKO (black square, dashed line) and controls (white circle, solid line). * indicates significant difference at p<0.05.

To determine the effects of muscle-specific GHR disruption on glucose homeostasis, we measured fasting blood glucose, plasma insulin, c-peptide, adiponectin and glucose tolerance at 6 months of age (n=15-17, per group per sex). Fasting blood glucose (Fig 3A) was significantly decreased in male MuGHRKO mice compared to controls; however, fasting blood glucose levels in female MuGHRKO mice did not differ from controls. Similarly, fasting levels of insulin (Fig 3B) and c-peptide (Fig 3C) were significantly decreased in males, while insulin and c-peptide levels in female MuGHRKO were unchanged. Circulating adiponectin levels were significantly decreased in females, but unchanged in male MuGHRKO mice (Fig 3D). Glucose tolerance was significantly improved in MuGHRKO males (Fig 3E) but not in females (Fig 3F). Thus, the hyperglycemic tendency in glucose homeostasis in males is improved when GH action is disrupted specifically in muscle.

### Muscle-specific GHR disruption has opposite effects on body weight in males and females

**Figure 4:**
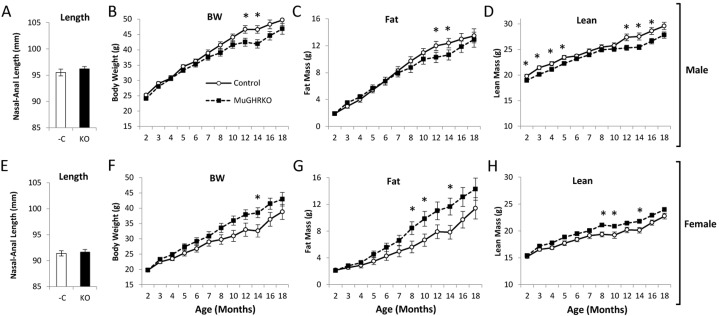
Body length and longitudinal body composition in MuGHRKO mice Nasal-anal body lengths are shown for (**A**) male and (**E**) female MuGHRKO (black bars) and controls (white bars). (**B**,**F**) Body weights, (**C**,**G**) fat mass, and (D,H) lean mass are indicated for male and female MuGHRKO (black square, dashed line) and controls (white circle, solid line). * Indicates significant difference at p<0.05.

In order to assess the effects of muscle-specific disruption of GH action on body size and composition, body lengths were determined at 6 months of age (n=14-16, per group per sex), and body weight and body composition were determined over time from 2 to 18 months of age (n=17-22, per group per sex). Body lengths were unchanged in MuGHRKO mice compared to controls for both sexes (Fig 4). Body weight was altered in opposite directions in male vs female MuGHRKO mice compared to same sex controls. Male MuGHRKO mice had decreased body weight reaching statistical significance at 12 and 14 months of age. In contrast, female MuGHRKO mice had increased body weight at 14 months of age. Similarly fat mass was decreased at 12 and 14 months in male MuGHRKO mice, but was increased in females at 8, 10, and 14 months. Lean mass was also altered sex-specifically with male MuGHRKO mice displaying decreased lean mass that reached statistical significance at 2-5 months of age and again at 14-16 months of age, while female MuGHRKO mice had increased lean mass that was statistically significant at 8, 10, and 14 months of age. Thus, direct GH action on muscle has mild but opposite effects on body weight depending on sex.

### Energy expenditure and musculoskeletal frailty are unaltered in old MuGHRKO mice

**Figure 5:**
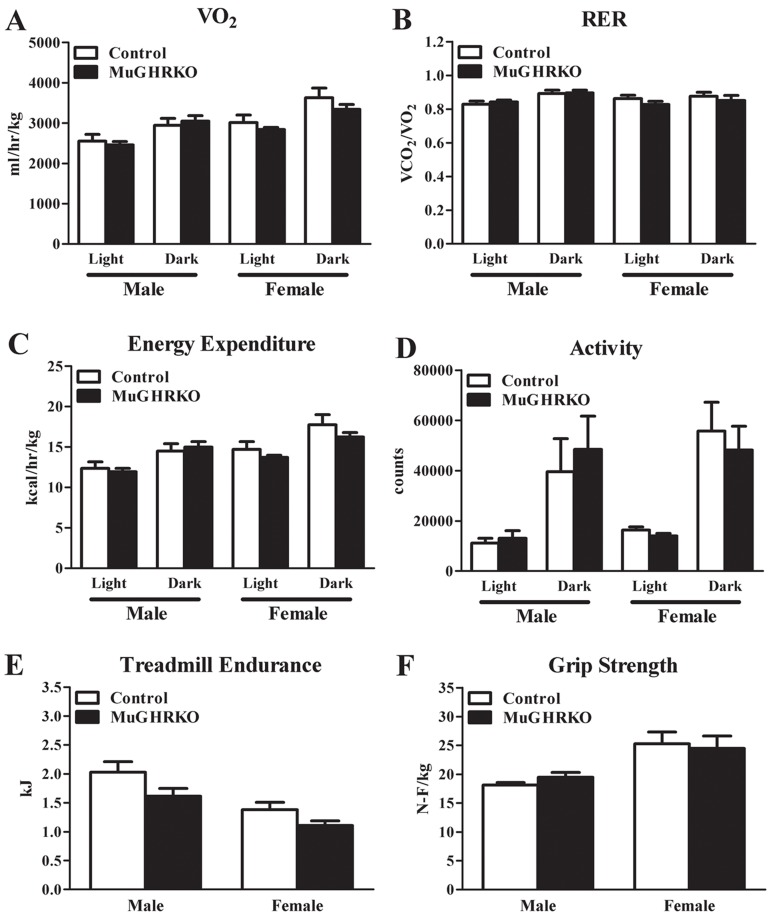
Indirect calorimetry, endurance, and grip strength (**A**) Day and night values for VO_2_, (**B**) respiratory exchange ratio, (**C**) energy expenditure, and (**D**) activity are shown for MuGHRKO (black bars) and controls (white bars) for males and females. (**E**) Treadmill endurance and (**F**) forelimb grip strength values are shown for MuGHRKO and controls. All values are represented as mean + SEM (n=6-8 per group per sex).

To determine the effects of muscle-specific GHR disruption on energy expenditure as well as musculoskeletal frailty in males and females, we evaluated indirect calorimetry measures, grip strength, and treadmill endurance in aged (2 year old; n=6-8, per group per sex) mice. Day and night values for VO_2_ normalized to lean body mass did not differ between MuGHRKO and controls (Figure 5A). Similarly, MuGHRKO mice of both sexes showed no change in respiratory exchange ratio (Figure 5B), no change in energy expenditure normalized to lean body mass (Figure 5C), and no change in activity (Figure 5D) compared to controls. Treadmill endurance and forearm grip strength were also similar in MuGHRKO mice compared to controls of both sexes (Figure 5E). Other measures, such as food intake adjusted for body weight, did not differ between MuGHRKO and controls in either sex (data not shown). Thus, direct GH action on muscle has no substantial effect on metabolism or musculoskeletal frailty.

### Evaluation of lifespan at Ohio University and the University of Michigan in male and female MuGHRKO mice

**Figure 6:**
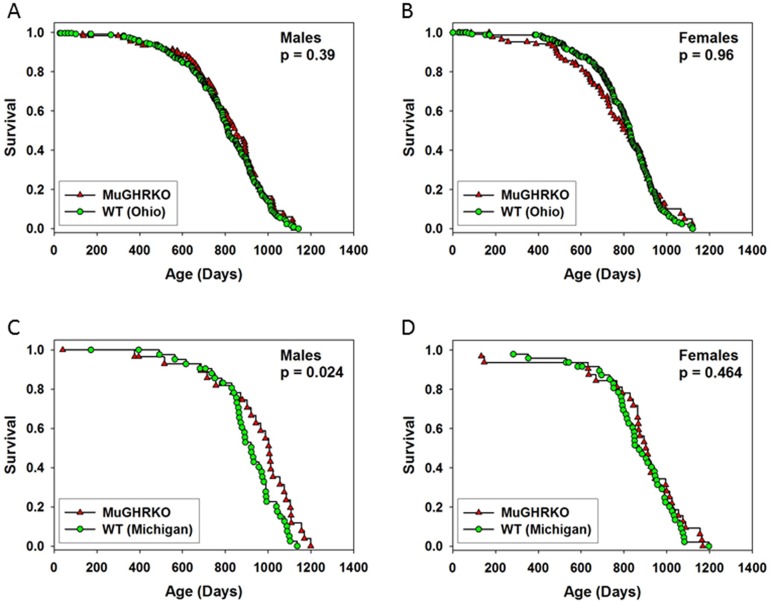
Effects of muscle-specific disruption of GHR on longevity (**A**-**B**) Survival tables for MuGHRKO (red triangles) and control mice (green circles) at Ohio University (**C**-**D**) Survival of MuGHRKO and control mice at the University of Michigan P values are derived from the log-rank test; p > 0.05 was considered significant.

Lifespan was evaluated in two independent experiments, one done at Ohio University (OU) and the other at the University of Michigan (UM). At OU, the initial cohort included 536 control mice and 230 MuGHRKO mice, with approximately equal numbers of each sex. Of these 766 mice, 291 were removed from the colony for other experimental purposes (treated as lost to follow up at the day of removal), and 475 died at the end of their natural lifespan. There were no significant differences between MuGHRKO and control mice at OU in either sex (Figure 6, panel A and B). The control mice at OU included 150 that had the Cre allele only, 216 with the Floxed allele only, and 230 with neither Cre nor Flox; there were no significant differences in longevity among these three control groups (not shown). At UM, the initial cohort included 62 MuGHRKO mice (32 female) and 93 control mice (48 female). The control group consisted of mice homozygous for the Floxed allele of GHR but lacking Cre. These controls were derived from three breeding stocks, one segregating Cre under the control of the albumin promoter [LiGHRKO; see [22]], one with Cre controlled by the aP2 promoter [FaGHRKO; see [26]], and one segregating Cre driven by the muscle creatine kinase promoter (MuGHRKO, this paper). There were no significant differences among the three varieties of control mice, which were thus pooled to a single control group. As shown in Figure 6, panel C, MuGHRKO males were significantly longer-lived than control mice (p = 0.02). Median survival increased from 922 days to 1004 days, an increase of 9%. The age at 90th percentile mortality increased from 1089 to 1156 days (6% increase). The Wang/Allison test [32] was used to estimate the likelihood that the two groups differed in the percent of live mice at the age (1104 days) at which 90% had died in the pooled survival table; this gave p = 0.03 in a two-sided test, showing a significant effect on this surrogate for maximum lifespan. MuGHRKO females did not show any effect on lifespan at UM (Figure 6, panel D). When the data for males and females were combined, the log-rank test gave p = 0.04, showing increased lifespan in MuGHRKO mice, but the Wang/Allison test failed to show a significant effect on 90th percentile survival (p = 0.08; data not shown).

End-of-life pathology was evaluated for 244 of the mice from the colony at OU, with the pathologists providing a best estimate of the likely cause of death. Results are shown in Table 1. Approximately 81% of male control and 91% of female control mice died from neoplastic diseases (includes cases in which death was due to multiple causes, i.e., neoplastic & non-neoplastic diseases). The major fatal neoplastic diseases observed in these control mice were lymphoma, hepatocellular carcinoma (HCC), pulmonary adenocarcinoma, and pituitary adenoma. In addition, the neoplastic diseases were usually associated with metastases to the other organs or other pathological lesions, e.g., pleural effusion, ascites, hemorrhage in the pleural and/or abdominal cavities, and severe congestion and edema in the lung. The incidence of fatal neoplasms in male (approximately 92%) and female (approximately 75%) MuGHRKO mice was similar to controls. Incidence of each neoplastic disease was similar between MuGHRKO and control mice (both male and female).

The major, fatal non-neoplastic diseases observed in these mice were glomerulonephritis and thrombus in the heart. These lesions were usually associated with other pathological lesions, e.g., pleural effusion, ascites, and/or severe congestion and edema in the lung. The incidence of fatal glomerulonephritis was slightly lower in male MuGHRKO and slightly higher in female MuGHRKO mice than in the control group, but the difference was not statistically significant. Thus, removing the direct action of GH in muscle does not lead to major changes in end-of-life pathology.

## DISCUSSION

Mutations that improve longevity may do so by targeting fundamental aging processes. This, in turn, may delay onset or progression of age-related dysfunction and diseases. Therefore, understanding the effects of these mutations may prove useful for learning about the pathways that lead to, or mitigate, long-term health and late-stage disease. While many null mutations can decrease lifespan in mice, a few have been shown to increase lifespan. One such example is disruption of the GHR gene. Global GHR−/− mice are exceptionally long-lived and have improved glucose homeostasis and resistance to diabetes and cancer [6]. While several mechanisms have been proposed to explain why global GHR−/− mice are long-lived, improved glucose homeostasis remains a likely candidate. It has long been established that GH has anti-insulin (also known as diabetogenic) activity. Because GLUT4, the only insulin-responsive glucose transporter, is present primarily in muscle (skeletal and cardiac) and adipose tissue (brown and white) and because muscle is responsible for the bulk of insulin stimulated glucose uptake (approximately 4x that of adipose tissue)[33], we tested the hypothesis that loss of direct GH action on muscle might contribute to the improved longevity in global GHR−/− mice. We found that disrupting the GHR gene in muscle improves glucose homeostasis in males, with decreased fasting blood glucose, decreased insulin, and improved glucose tolerance. Female MuGHRKO mice had fasting blood glucose, insulin, and glucose tolerance that were similar to control females and consistent with a pattern of superior insulin sensitivity, compared to males of either genotype. This sexual-dimorphic improvement to glucose homeostasis may be explained by the difference in baseline glucose homeostasis in the control mice in that further improvements may be more difficult to achieve in the relatively insulin sensitive female mice. Increased mean and maximal lifespan was detected in male MuGHRKO mice in at least one cohort, at UM. However, this finding was not consistent since longevity in male MuGHRKO mice at OU did not significantly differ from controls. Histopathological analysis revealed that causes of death in MuGHRKO mice were essentially unchanged, with similar rates of cancer and glomerulonephritis compared to controls, potentially reflecting the lack of alteration in IGF-1 levels when the GHR gene is disrupted in muscle. Finally, measures of musculoskeletal frailty and metabolism were unchanged with advanced age, suggesting that the direct action of GH on muscle has minimal effect on strength and endurance during aging.

Aside from caloric restriction (CR), disruption of the GH/IGF-1 axis is perhaps the most robust intervention to increase lifespan in mice. Many studies have shown that alterations in the GH/IGF-1 axis can affect lifespan [4]. For example, GH transgenic mice, with increased GH action, have dramatically decreased lifespans with numerous indicators of poor health [4]. On the other hand, mice with decreased GH action have been shown to live longer and have delays in occurrence and progression of age related disease and functional impairment [4, 6]. Global GHR−/− mice, which are particularly informative because their perturbations are restricted to the GH/IGF-1 axis, are long-lived and have partial overlap with phenotypic characteristics of calorically restricted (CR) mice. Among these shared traits are reduced serum IGF-1 levels [6], reduced cellular senescence in white adipose tissue [10], decreased mTOR activity [34], protection from musculoskeletal frailty with better retention of strength, balance, and motor coordination in old age [9], reduced rates and delayed occurrence of cancer [15], and enhanced glucose homeostasis with reduced levels of circulating insulin [4]. In the current study, we show that muscle-specific disruption of GHR significantly improves glucose tolerance and decreases insulin levels in male MuGHRKO mice, but not in females. An increase in survival and in maximal lifespan was detected in male MuGHRKO at UM mice, though not in a parallel experiment at OU and not in females at either test site. It is not known why extended longevity in male MuGHRKO mice was observed at UM but not at OU; however, small increases in lifespan are difficult to replicate from institution to institution [35]. Slight differences among colonies in animal husbandry and environment are unavoidable, including subtle differences in room temperature, noise levels, lighting, air pressure, and laboratory animal personnel. Ultimately, due to the variable findings at the two sites, we cannot with certainty conclude if removal of GHR in male mice improves longevity. However, we can conclude that there is not a negative effect on lifespan.

While removal of GH action in muscle of male mice results in features that are consistent with the hypothesis that blocking the anti-insulin activity of GH improved glucose homeostasis, the hypothesis that improved glucose homeostasis in MuGHRKO mice will improve lifespan remains questionable. However, we do know that removal of GHR in muscle did not shorten lifespan as discussed above. Since MuGHRKO mice were one of three lines simultaneously generated and studied by our laboratories, we can compare the effects of disrupting GHR in three insulin sensitive tissues (muscle, liver, and fat). Our previous work with liver- and fat-specific GHR gene disrupted mice indicates that lifespan does not always positively correlate with glucose homeostasis. For example, liver-specific disruption of the GHR (LiGHRKO) produces mice that have impaired glucose homeostasis [21, 22]. However, these mice have a normal lifespan as determined by two laboratories (OU and UM)[34]. Furthermore, fat-specific disruption of GHR (FaGHRKO) produces mice that have normal glucose homeostasis and these mice are short lived (List, Kopchick and Miller unpublished results at OU and UM). This suggests that other processes related to aging may have been altered (improved in LiGHRKO and impaired in FaGHRKO mice) to counteract the effect of glucose homeostasis on aging. For example, when GHR is disrupted in liver, mechanisms other than improved glucose homeostasis (such as decreased circulating IGF-1) may protect LiGHRKO mice from decreased lifespan. Taken together, combined longevity results for liver-, fat, and muscle-specific GHR gene disrupted mice suggest that multiple mechanisms account for improved longevity in global GHR−/− mice, and these mechanisms can vary dramatically depending on the tissues that are disrupted. Overall, it appears that these mechanisms are strongly based on the overarching reduction in the IGF/insulin axis as found in the GHR−/− mice. As discussed earlier, this effect shows substantial overlap with calorie restriction models, which also have a reduction in serum IGF-1/insulin levels and share many of the traits seen in GHR−/− mice.

The sexual-dimorphic improvement to glucose homeostasis in male MuGHRKO mice may be explained by baseline glucose homeostasis in the control mice. More specifically, female control mice in the C57BL/6J background have lower insulin and glucose tolerance compared to male control mice, as we show in this study and has been previously reported by others [36, 37]. In fact, control female mice in this strain show protection from alterations in glucose homeostasis even when challenged with high fat feeding [38]. Thus, it is possible that the reason that female muscle GHR disrupted mice do not have improvements in lifespan could be that no further improvement in glucose homeostasis is possible in the relatively insulin sensitive female mice. Obviously, these sex-related differences in insulin sensitivity of control mice could be attributed, in part, to actions of estrogen and testosterone. Indeed, estrogen has been shown to increase phosphorylation of insulin receptor substrate and Akt [39] and activate GLUT-4 and glucose uptake into muscle [40] in other model systems. However, estrogen also influences adipose tissue development and function, which could also account for the improved insulin sensitivity in female groups [41]. It may be of interest in future studies to consider ovariectomizing female MuGHRKO mice to confirm the importance of estrogen in this sexual dimorphic response.

Prior to the current study, two separate lines of muscle-specific GHR gene disrupted mice had been generated; however, conflicting results were reported and only males had been studied [27, 28]. Mavalli and colleagues [27] report that muscle specific disruption of GHR in male mice produces increased adiposity with insulin resistance, and glucose intolerance. In contrast, Vijayakumar and colleagues report reduced adiposity and overall improvement in glucose homeostasis [28]. The differences in results between the two laboratories likely reflect the use of different promoter/enhancers driving Cre expression in the two mouse lines. Vijayakumar et al. used muscle creatine kinase (Ckmm) promoter/enhancer [42], which drives Cre expression in postnatal skeletal and cardiac muscle [28, 43]. This promoter is identical to what we used, and our results in male mice in terms of glucose homeostasis are similar. Mavalli et al. used the mef-2c promoter, which directs Cre expression in postnatal skeletal muscle [44]. While mef-2c Cre expression was thought to target skeletal muscle exclusively, recently it has been shown that it is an important regulator of brain, bone, lymphocyte, blood vessel, endothelium, neural crest, craniofacial, and melanocyte development [45, 46]. Thus, unanticipated expression of Cre by the mef-2c promoter in tissues other than muscle may explain the differences between the two mouse lines.

In addition to being long lived, global GHR−/− mice are protected from musculoskeletal frailty suggesting that GH action over time eventually results in deterioration of the musculoskeletal system [9]. More specifically, global GHR−/− mice have superior grip strength, balance, and motor coordination at advanced ages. This protection from frailty resulting from decreased GH action is not limited to global GHR−/− mice as Ames dwarf mice are also resistant to age related declines in musculoskeletal performance [47]. Whether this protection results from removing direct GH action on skeletal muscle or is due to the cumulative, indirect effects of GH action in these dwarf lines is not established since the disruptions to GH action is global in these lines. In the current study, MuGHRKO mice show no protection from normal age related decline in grip strength or treadmill endurance. Thus, the protection from age-related loss in muscle strength, seen in global GHR−/− and Ames dwarf mice, seems to reflect GH actions mediated indirectly through other tissues, for example by alterations in IGF-1 production in liver. Since caloric restriction (CR) also results in protection from musculoskeletal frailty [9, 47], the shared phenotypes of these two interventions (reduced GH action and CR) suggest the cause may be more likely due to decreased IGF-1. Data on liver-specific GHRKO mice (LiGHRKO) are consistent with the notion that reduced IGF-1 (and not reduced GH on muscle) is responsible for protection against age-related loss of muscle function in these mice. LiGHRKO mice have a ~90% reduction in IGF-1 with elevated GH and retain relatively high grip strength at advanced age, in both sexes, compared to controls. Since only GH action in the muscle is disrupted in MuGHRKO mice and since these mice are not protected from frailty, direct GH action on muscle does not appear to contribute to musculoskeletal frailty. Collective data regarding muscle from MuGHRKO, global GHR−/−, Ames, and LiGHRKO mouse lines suggests that removing the indirect GH action, i.e. lowering IGF-1, may be more important in protection against musculoskeletal frailty.

In conclusion, this study provides evidence concerning the specific loss of GH action on mouse muscle. There are several key findings that will serve as a guide for future studies. First, these data demonstrate that male MuGHRKO mice have improvements in glucose homeostasis, while females show no further improvements. Second, while global disruption of GHR has been shown to protect against musculoskeletal frailty, surprisingly, removing the direct action of GH in muscle shows no effect. Thus, indirect actions of GH on muscle appear to be responsible for this protection. When considered with results from LiGHRKO mice, perhaps IGF-1 may be responsible for this protection. Third, when taken together, combined longevity results for liver-, fat, and muscle-specific GHR gene disrupted mice suggest that multiple mechanisms account for improved longevity in global GHR−/− mice and these mechanisms can vary dramatically depending on the tissues that are disrupted.

## METHODS

### MuGHRKO mice

Conditional GHR “floxed” mice were generated as described previously [26]. Muscle-specific GHR knockout mice (MuGHRKO) (FFCx) and floxed littermate controls (FFxx) were produced by crossing floxed GHR mice produced in a C57BL/6N background to B6.FVB(129S4)-Tg(Ckmm-cre)5Khn/J mice (Cat# 006475; Bar Harbor, ME), which was previously backcrossed into the C57BL/6J strain. Therefore, the resulting MuGHRKO mice were a mix of C57BL/6J and C57BL/6N substrains. Breedings were coordinated in such a manner that all mice were C57BL/6 with a 62.5% “J” and 37.5% “N” substrain mixture at Ohio University. Breeding pairs were shipped from Ohio University to the University of Michigan where they were maintained in the same C57BL/6 (62.5% J/37.5% N) substrain mixture for determination of longevity. Mice were housed 2-4 per cage and given ad libitum access to water and standard laboratory rodent chow in which 14% of energy is from fat, 60% from carbohydrates, and 26% from protein (5P00 ProLab RMH 3000; LabDiet, St. Louis, MO, USA). The cages were maintained in a temperature-controlled room (22°C) and exposed to a 14-hour light/10-hour dark cycle. All procedures were approved by the Ohio University Institutional Animal Care and Use Committee and by the University of Michigan University Committee on the Use and Care of Animals. For lifespan analyses, cages were inspected daily. Date of death was noted and mice found to be so ill that they were expected to die within the next 24–48 h were euthanized with the date of euthanasia taken as the date of death for life table calculations. Mice were removed from the study because of fighting, accidental death, or ulcerative dermatitis, or because they were used for another experimental purpose, such as dissection or shipped to Mayo Clinic for indirect calorimetry analysis. For survival analyses, all such mice were treated as alive at the date of their removal from the protocol and lost to follow-up thereafter.

### Muscle-specific disruption of GHR

Muscle-specific disruption of the GHR gene was verified by PCR in WAT (subcutaneous, epididymal, retroperitoneal, and mesenteric depots), BAT, liver, skeletal muscles (quadriceps, soleus, gastrocnemius), heart, kidney, spleen, and brain tissue collected from LiGHRKO and floxed control mice. DNA isolated from global GHR gene disrupted (EIIa) mice and floxed littermate controls were used as positive and negative controls, respectively. PCR was performed as previously described [22, 26]. Relative expression of GHR mRNA was determined by real time RT-PCR as previously described [22, 26].

### Circulating factors, fasting blood glucose and glucose tolerance tests

Starting at 9 am after an overnight 12 hour fast, 64 mice (n=15-16 per group) were sacrificed at 6 months of age. Just prior to dissection, serum was collected from the orbital sinus. Serum GH, IGF-1, IGFBPs, insulin, c-peptide, and adiponectin were measured as previously described [22]. Blood glucose levels were determined at 5 months of age using a OneTouch Ultra glucometer and test strips (Lifescan Inc. Milpitas, CA, USA). Blood samples were obtained from the tip of the tail, and glucose tolerance tests were performed between 5-6 months of age as previously described [26]. Briefly, for GTTs, 12 hour overnight fasted mice received an intraperitoneal injection of 10% glucose at a dose of 1g/kg body weight at ~9:00 am. Blood glucose measurements were determined before the glucose injection (time 0) and at 15, 30, 45, 60, and 90 minutes post injection. Mice were given at least 1 week between fasting blood glucose and GTTs.

### Body composition and body length determination

Body composition was determined over time starting at 2 months until 18 months of age (n=15-22 per group) using a Bruker Minispec mq NMR analyzer (The Woodlands, TX, USA) as previously described [22, 26, 48]. Nasal-anal body length was determined at 6 months of age (n=15-16) in euthanized mice just prior to dissection.

### Indirect calorimetry data and age related frailt

Two year old male and female MuGHRKO and control mice were sent to Mayo Clinic for indirect calorimetry and analysis of grip strength and treadmill endurance as previously described [22].

### Pathological examination

Mice that were sacrificed or died spontaneously were removed from the cage, immediately necropsied with gross pathological examination, and preserved in 10% buffered formalin. After the mice were examined for gross pathological lesions, the following organs and tissues were excised and fixed with Bouin's solution: brain, pituitary gland, heart, lung, trachea, thymus, aorta, esophagus, stomach, small intestine, colon, liver, pancreas, spleen, kidneys, urinary bladder, reproductive system (male: prostate, testes, epididymis, seminal vesicles; female: ovaries, uterus), thyroid gland, adrenal glands, parathyroid glands, psoas muscle, knee joint, sternum, and vertebrae. Any other organ or tissue in which lesions were observed by gross inspection was excised. The fixed tissues were embedded in paraffin, sectioned at 5 μm, and stained with hematoxylin-eosin. Diagnosis of each histopathological change was made using histological classifications for aging mice, as previously described [49-51]. Two pathologists separately examined the samples without knowledge of their genotype or age. The probable cause of death was determined independently by the two pathologists based on the severity of the pathology found at necropsy. In more than 90% of the cases, there was agreement by the two pathologists. In cases where the two pathologists did not agree or where disease did not appear severe enough, the cause of death was categorized as unknown.

### Statistical analysis

Unless otherwise stated, means ± SE are presented in the figures, and significance was determined by a Student t-test. A P value of < 0.05 was considered statistically significant. Survival curves were compared using the log-rank test. All significance tests about survival are based upon the two-tailed log-rank test at P < 0.05, with censored mice included up until their date of removal from the longevity population. For pathology, the total frequency and grade of lesions were compared between genotypes using a chi-square test [52]. When the expected frequencies were too small for the chi-square test, data were analyzed using Fisher's exact test [52]. Fatal neoplastic lesions and adenocarcinoma were analyzed using a log-rank test [53].
